# Structural Features of Ion Transport and Allosteric Regulation in Sodium-Calcium Exchanger (NCX) Proteins

**DOI:** 10.3389/fphys.2016.00030

**Published:** 2016-02-09

**Authors:** Moshe Giladi, Inbal Tal, Daniel Khananshvili

**Affiliations:** Department of Physiology and Pharmacology, Sackler Faculty of Medicine, Tel Aviv UniversityTel Aviv, Israel

**Keywords:** NCX, allosteric regulation, Ca^2+^ binding proteins, X-ray crystallography, HDX-MS, SAXS

## Abstract

Na^+^/Ca^2+^ exchanger (NCX) proteins extrude Ca^2+^ from the cell to maintain cellular homeostasis. Since NCX proteins contribute to numerous physiological and pathophysiological events, their pharmacological targeting has been desired for a long time. This intervention remains challenging owing to our poor understanding of the underlying structure-dynamic mechanisms. Recent structural studies have shed light on the structure-function relationships underlying the ion-transport and allosteric regulation of NCX. The crystal structure of an archaeal NCX (NCX_Mj) along with molecular dynamics simulations and ion flux analyses, have assigned the ion binding sites for 3Na^+^ and 1Ca^2+^, which are being transported in separate steps. In contrast with NCX_Mj, eukaryotic NCXs contain the regulatory Ca^2+^-binding domains, CBD1 and CBD2, which affect the membrane embedded ion-transport domains over a distance of ~80 Å. The Ca^2+^-dependent regulation is ortholog, isoform, and splice-variant dependent to meet physiological requirements, exhibiting either a positive, negative, or no response to regulatory Ca^2+^. The crystal structures of the two-domain (CBD12) tandem have revealed a common mechanism involving a Ca^2+^-driven tethering of CBDs in diverse NCX variants. However, dissociation kinetics of occluded Ca^2+^ (entrapped at the two-domain interface) depends on the alternative-splicing segment (at CBD2), thereby representing splicing-dependent dynamic coupling of CBDs. The HDX-MS, SAXS, NMR, FRET, equilibrium ^45^Ca^2+^ binding and stopped-flow techniques provided insights into the dynamic mechanisms of CBDs. Ca^2+^ binding to CBD1 results in a population shift, where more constraint conformational states become highly populated without global conformational changes in the alignment of CBDs. This mechanism is common among NCXs. Recent HDX-MS studies have demonstrated that the apo CBD1 and CBD2 are stabilized by interacting with each other, while Ca^2+^ binding to CBD1 rigidifies local backbone segments of CBD2, but not of CBD1. The extent and strength of Ca^2+^-dependent rigidification at CBD2 is splice-variant dependent, showing clear correlations with phenotypes of matching NCX variants. Therefore, diverse NCX variants share a common mechanism for the initial decoding of the regulatory signal upon Ca^2+^ binding at the interface of CBDs, whereas the allosteric message is shaped by CBD2, the dynamic features of which are dictated by the splicing segment.

## Introduction

Calcium (Ca^2+^) is the most important and versatile secondary messenger in the cell; it carries vital information to virtually all processes important to cell life and function (e.g., it couples excitation to contraction, hormone secretion, gene transcription, and controls enzyme activity through protein phosphorylation-dephosphorylation involving numerous biochemical reactions). The evolutionary choice of Ca^2+^ as a universal and versatile intracellular messenger has been dictated by its coordination chemistry (Williams, 1999), although how these chemical properties of Ca^2+^ are realized in protein-calcium interactions and how this is translated to biological functions of diverse Ca^2+^-binding proteins are currently not entirely clear (Gifford et al., 2007). Since Ca^2+^ promotes, maintains, and modifies the programmed function and demise of various cell types by governing numerous signal transduction pathways (Carafoli, 1987; Berridge et al., 2003), it is not surprising that an altered handing of Ca^2+^ homeostasis can precipitate disease-related conditions.

Maintenance of resting cytosolic Ca^2+^ levels (~100 nM) is essential in every living cell, where the maintenance of resting cytosolic levels as well as the cell-specific dynamic oscillations of cytosolic Ca^2+^ requires tight regulation and integration of Ca^2+^ transport proteins. Notably, the Ca^2+^ transporting proteins are located in the plasma membrane and in the membranes of the organelles (the endo/sarcoplasmic reticulum, the mitochondria, and the nuclear envelope), thereby playing distinctive roles in the excitation-contraction coupling of cardiac (Bers, 2002) and skeletal (Melzer et al., 1995) muscle cells, the release of neurotransmitters (Neher and Sakaba, 2008), apoptosis (Orrenius et al., 2003), mitochondrial bioenergetics (Filadi and Pozzan, 2015), among others. These oscillations must occur in the right place and the right time to fulfill functional requirements in diverse cell types (e.g., excitable tissues) (Carafoli, 1987; Bers, 2002; Berridge et al., 2003; Brini et al., 2014).

**Figure 1:**
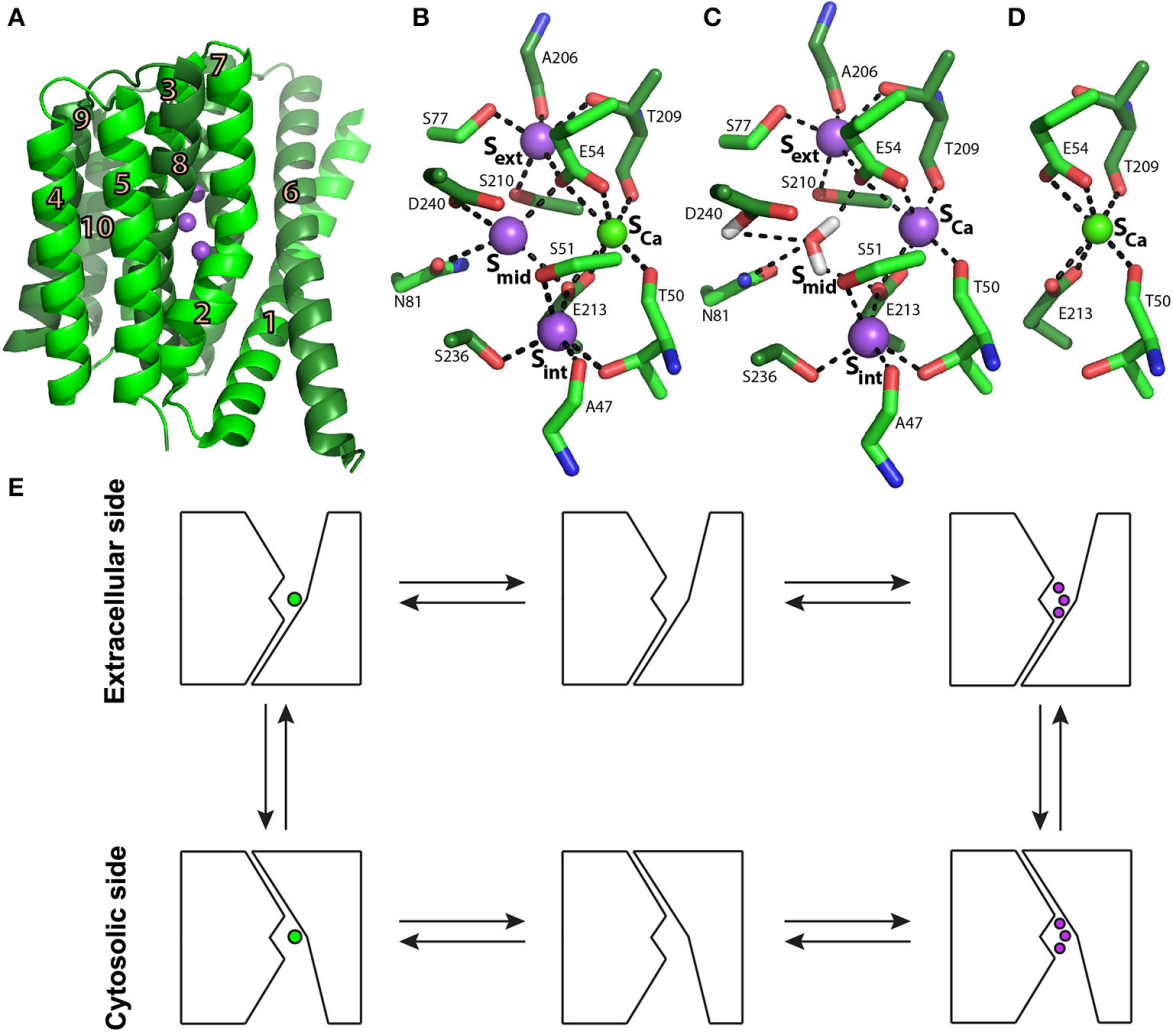
**NCX_Mj structure and transport mechanism**. **(A)** Crystal structure of NCX_Mj (PDB 3V5U) in cartoon representation. Helices 1-5 (TM1-5) are light green and helices 6-10 (TM6-10) are dark green. Purple and green spheres represent the Na^+^ and Ca^2+^ ions, respectively. **(B)** Ion coordination, as suggested by the crystal structure of NCX_Mj. **(C)** 3Na^+^ ion coordination, as suggested by molecular dynamics simulations and ion-flux assays. **(D)** Ca^2+^ binding site. **(E)** Schematic representation of the ion-exchange mechanism.

The multifaceted effects of Ca^2+^ signaling pathways require dynamic regulation, coordination, and the integration of ion channels, pumps, and transporters involved in Ca^2+^ transport, buffering, and storage (Carafoli, 1987; Berridge et al., 2003). The PM (plasma membrane) Ca^2+^-ATPase, and Na^+^/Ca^2+^ exchanger (NCX) extrude Ca^2+^ from the cell, although their partial contributions to Ca^2+^ homeostasis differ among distinct cell types, depending on the functional specialization and regulatory specificity in a given cell type (Khananshvili, 2013, 2014; Brini et al., 2014). For example, in cardiomyocytes, NCX serves as a high-capacity (*k*_cat_ ~ 2500 s^−1^) and low-affinity (K_m_ ~ 10–20 μM) system that allows rapid removal of Ca^2+^; it can limit Ca^2+^ transients over a wide dynamic range (Carafoli, 1987; Bers, 2002; Khananshvili, 2013, 2014). NCX proteins mediate uphill Ca^2+^-fluxes in exchange with downhill Na^+^ transport, with a stoichiometry of 3Na^+^:1Ca^2+^ (Figure 1), thus creating an electrogenic current (Reeves and Hale, 1984). NCX works mainly in the forward mode, i.e., it extrudes Ca^2+^ from the cell. However, under certain altered conditions (e.g., high intracellular Na^+^, highly positive membrane potential) NCX may work in the reverse mode and induce Ca^2+^ influx (Blaustein and Lederer, 1999). Three mammalian NCX genes (*SLC8A1, SLC8A2*, and *SLC8A3*) and their splice variants are expressed in a tissue-specific manner (Philipson and Nicoll, 2000). By regulating cytosolic [Ca^2+^], the protein products (NCX1, NCX2, and NCX3, respectively) modulate fundamental physiological events, such as muscle excitation-contraction coupling, neuronal long-term potentiation and learning, blood pressure regulation, immune responses, neurotransmitter and insulin secretion, and mitochondrial bioenergetics (Khananshvili, 2013, 2014; Filadi and Pozzan, 2015). Altered expression and regulation of NCXs actively contribute to distorted Ca^2+^-homeostasis, resulting in molecular and cellular remodeling of distinct tissues, which is associated with pathophysiological states including heart failure, arrhythmia, cerebral ischemia, hypertension, diabetes, renal Ca^2+^ reabsorption, and muscle dystrophy, among others. Thus, NCX proteins represent a long-wanted target for selective pharmacological targeting (Khananshvili, 2014).

Structurally, eukaryotic NCX proteins are composed of 10 transmembrane (TM) helices and contain a large cytosolic regulatory loop (f-loop) between TM5 and TM6 (Ren and Philipson, 2013). The major difference between eukaryotic and prokaryotic NCX proteins is that prokaryotic NCXs lack the large f-loop, which includes two regulatory Ca^2+^-binding domains, CBD1 and CBD2 (Hilge et al., 2006; Liao et al., 2012). In eukaryotes, these regulatory domains enable the dynamic adjustment of Ca^2+^-extrusion rates from the cell in accordance with the dynamic oscillations of cytosolic Ca^2+^, representing a regulatory feedback mechanism (Blaustein and Lederer, 1999; Philipson and Nicoll, 2000; Hilge et al., 2006). The dynamic regulation of NCX is especially diverse and complex, since it must remove large amounts of Ca^2+^ within a limited time window. Ca^2+^-extrusion rates via NCX must change within milliseconds to match the dynamic oscillation in the cytosolic Ca^2+^, i.e., during the action potential in cardiomyocytes (Berridge et al., 2003; Boyman et al., 2011). Ca^2+^ interaction with the regulatory CBDs (located 70–80 Å away from the transport sites) of cardiac NCX enhances the turnover rates of NCX up to 25-fold (Boyman et al., 2011), where Ca^2+^ extrusion rates dynamically change in response to dynamic changes in the membrane potential and the cytosolic Na^+^ and Ca^2+^ concentrations during the action potential.

Over the last decade, diverse structural methods, including nuclear magnetic resonance (NMR), X-ray crystallography, small angle X-ray scattering (SAXS), fluorescence resonance energy transfer (FRET) and hydrogen-deuterium exchange mass spectrometry (HDX-MS) have been utilized to study the mechanisms underlying ion transport and the allosteric regulation of NCX. Despite the tremendous progress, some important questions remain open: Why are so many isoforms and splice variants required by different cell types and why does each cell type express a specific set of isoforms and splice variants? What are the exact mechanisms underlying the function and regulation of diverse isoforms and splice variants? What is the partial contribution of distinct splice variants to specific functions in a given cell type? During the last few years, huge progress has been made in better understanding the molecular mechanisms underlying NCX regulation in tissue-specific isoforms and splice variants. This review will focus on insights into NCX ion transport and allosteric regulation mechanisms derived from structural biology techniques in recent years.

## Crystal structure of an archaeal NCX as a prototype for the NCX ion transport mechanism

Biochemical studies utilizing transport assays in proteoliposomes (Khananshvili, 1990), followed by electrophysiological studies (Hilgemann et al., 1991; Niggli and Lederer, 1991), have concluded that NCX operates through a ping-pong mechanism in which one Ca^2+^ and three Na^+^ ions are translocated sequentially in separate steps rather than simultaneously across the membrane. This mechanism implies the alternating access mechanism of the NCX ion binding sites in the inward (cytosolic) and outward (extracellular) conformations (Figure 1E). A major advancement in better understanding the transport mechanism and ion selectivity was provided by solving the crystal structure of NCX from the archaebacterium *Methanococcus jannaschii* (NCX_Mj) (Liao et al., 2012).

The structure depicts NCX_Mj in the outward-facing conformation, composed of 10 transmembrane helices (TM1-10) with a pseudo molecular dyad (Figure 1A). It appears that this membrane topology is the same in mammalian NCX proteins (Ren and Philipson, 2013). As mentioned above, in sharp contrast with eukaryotic NCX, the cytosolic loop between TM5 and TM6 is extremely short (only 12 residues) in NCX_Mj, meaning that this loop cannot serve as a prototype for the large cytosolic regulatory f-loop of eukaryotic NCX.

The ion-binding pocket of NCX_Mj contains four ion-binding sites: S_ext_, S_mid_, S_int_, and S_Ca_(Figure 1B). The binding sites are arranged in a diamond-shaped configuration, where 12 residues contribute to Na^+^ and Ca^2+^ ligation (four in TM2 and TM7, and two in TM3 and TM8). Interestingly, 11 ion-coordinating residues (out of twelve) are highly conserved in organisms ranging from bacteria to humans, whereas in eukaryotic NCXs, D240 is consistently replaced by glutamine (Marinelli et al., 2014). Moreover, the ion exchange turnover rates increase nearly 10 times in the D240N mutant of NCX_Mj, thereby suggesting that the aspartate to asparagine replacement in eukaryotic species may represent an evolutionary “improvement” in catalytic power in mammalian NCX orthologs (Marinelli et al., 2014). The proximity and ligand sharing by the ions implicate the progressive antagonistic effect of Na^+^ binding on Ca^2+^ affinity and vice versa. In the outward conformation, the binding sites are exposed to high extracellular [Na^+^] levels, which favors Na^+^ binding and the release of Ca^2+^. When exposed to low intracellular [Na^+^] levels, Na^+^ release is favored, thus restoring the high Ca^2+^ affinity, which upon binding further decreases Na^+^ affinity (Liao et al., 2012).

According to the original interpretation of the crystallographic data, S_ext_, S_mid_, and S_int_ are occupied by 3Na^+^ ions, and S_Ca_ is occupied by one Ca^2+^ ion (Figure 1B) (Liao et al., 2012). However, the crystal structure of NCX_Mj revealed that this simultaneous occupation of all four sites by 3Na^+^ ions and one Ca^2+^ ion is thermodynamically forbidden (Marinelli et al., 2014). Recent molecular dynamics (MD) simulations and ion flux analyses revealed that 3Na^+^ ions occupy S_ext_, S_int_, and S_Ca_ (Figure 1C), whereas the Ca^2+^ ion occupies S_Ca_ (Figure 1D) (Marinelli et al., 2014). According to this interpretation, S_mid_ does not bind either the Na^+^ or Ca^2+^ ions and one water molecule is bound to protonated D240.

Eight helices of NCX_Mj (TM2-5 and TM7-10) generate a tightly packed hub (which is perpendicularly inserted into the membrane), whereas two long/slanting helices (TM1 and TM6) are loosely packed in front of the rigid eight-helix core (Figure 1A) (Liao et al., 2012). The sliding of the gating bundle (TM1/TM6) toward the rigid eight-helix core was proposed as a major conformational change that occurs during alternating access. Recently, three crystal structures of Ca^2+^/H^+^ exchangers were determined and revealed striking similarities with NCX_Mj, suggesting that the sliding mechanisms could be a general feature of the gene families belonging to the Ca/CA superfamily (Nishizawa et al., 2013; Waight et al., 2013). However, it remains unclear how ion binding drives the sliding of the gating bundle (the TM1/TM6 cluster) to initiate alternating access. The resolution of this mechanism is essential for understanding how Ca^2+^ binding to the regulatory CBDs in eukaryotic NCX orthologs accelerates the ion transport cycle.

## Allosteric regulation of eukaryotic NCX proteins

### Ionic regulation of NCX

NCX is allosterically regulated by its substrates, Ca^2+^ and Na^+^, and by H^+^ (Hilgemann, 1990; Boyman et al., 2011). A rise in [Na^+^]_i_ results in a decrease in NCX current in a dose-response manner, a process termed I_1_-inactivation or Na^+^-dependent inactivation (Hilgemann, 1990; Hilgemann et al., 1992b). A decrease in pH results in decreased NCX activity within a physiological and pathophysiological pH range (6.9–7.5) (Boyman et al., 2011). Interestingly, the effects of H^+^ and Na^+^ are interlaced: in the absence of Na^+^, pH has only a minor effect on NCX activity; under basic pH conditions, intracellular Na^+^ does not induce inactivation (Blaustein and Lederer, 1999).

**Figure 2:**
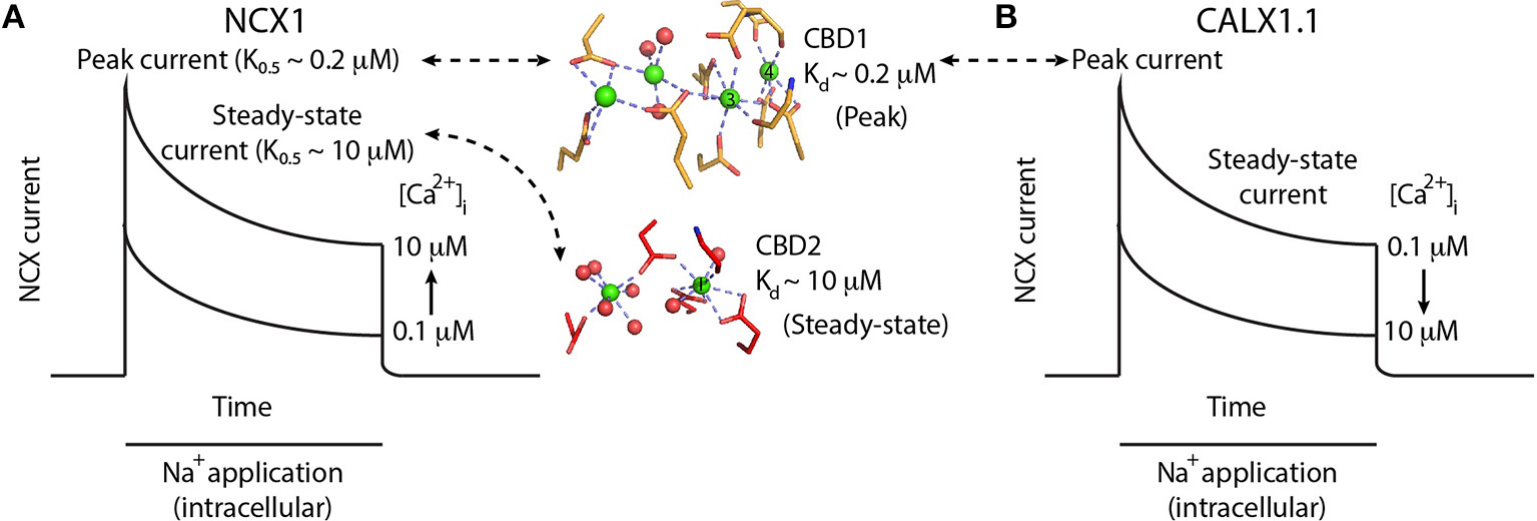
**Ca^2+^-dependent regulation of NCX and CALX. (A)** Schematic representation of NCX1 currents upon the application of intracellular Na^+^ to initiate the exchange reaction in the presence of varying [Ca^2+^]_i_. Na^+^ applications initially activates NCX1, followed by a slow decrease in exchange current representing Na^+^-dependent inactivation. Higher [Ca^2+^]_i_ results in larger peak currents and reduced Na^+^-dependent inactivation, as reflected by the higher steady-state current in the presence of 10 μM [Ca^2+^]_i_. Binding of Ca^2+^ ions (green spheres) to the Ca3-Ca4 sites of CBD1 (yellow sticks) results in peak-current activation, whereas the binding of one Ca^2+^ ion to the CaI site of CBD2 (red sticks) results in steady-state current activation. **(B)** Schematic representation of CALX1.1 currents upon the application of intracellular Na^+^ to initiate the exchange reaction in the presence of varying amounts of [Ca^2+^]_i_. Na^+^ application initially activates NCX, followed by a slow decrease in exchange current representing Na^+^-dependent inactivation. Higher [Ca^2+^]_i_ results in smaller peak currents and increased Na^+^-dependent inactivation, as reflected by the smaller steady-state current in the presence of 10 μM [Ca^2+^]_i_.

In contrast to Na^+^ and H^+^, a rise in [Ca^2+^]_i_ results in increased NCX current and counteracts the effect of [Na^+^]_i_(Hilgemann et al., 1992a). Moreover, regulatory cytoplasmic Ca^2+^ is obligatory for exchange activity (DiPolo, 1979). Removal of cytosolic Ca^2+^ results in slow inactivation of NCX, a process termed I_2_-inactivation or Ca^2+^-dependent inactivation (Hilgemann et al., 1992a). In patch-clamp recordings, a rise in NCX peak current represents [Ca^2+^]_i_-dependent activation of NCX, whereas the ratio between steady-state and peak currents represents the [Ca^2+^]_i_-induced relief of Na^+^-dependent inactivation (Figure 2A) (Hilgemann et al., 1992a). The Ca^2+^ sensitivity of the two processes is different: An increase in peak current occurs at lower [Ca^2+^] levels (~ 0.2 μM) than the increase in the steady-state to peak-current ratio (~ 10 μM) (Figure 2A) (Hilgemann et al., 1992a; Ottolia et al., 2009).

Interestingly, treatment of the intracellular surface of NCX with α-chymotrypsin abolished the regulatory effects of Na^+^, Ca^2+^, and H^+^ and resulted in constitutive NCX activation (Hilgemann, 1990; Matsuoka and Hilgemann, 1994). This finding demonstrates the existence of the ionic allosteric regulation of NCX through ions binding to one or more cytosolic regulatory domains that differ from those of the transport sites (Matsuoka et al., 1993).

### Alternative splicing and regulatory diversity of mammalian NCX proteins

In mammals, NCX1, NCX2, and NCX3 and their splice variants differ in their tissue-expression profiles—i.e., NCX1 is universally distributed, practically in every mammalian cell; NCX2 is expressed in the brain and spinal cord; and NCX3 is expressed in the brain and skeletal muscles (Philipson and Nicoll, 2000). At the post-transcriptional level, at least 17 NCX1 and 5 NCX3 splice variants are produced through alternative splicing of the primary nuclear *SLC8A1* and *SLC8A3* transcripts, whereas no splice variants have been identified for *SLC8A2* (Kofuji et al., 1994). Alternative splicing of NCX1 arises from combining six small exons (A, B, C, D, E, and F) located exclusively on CBD2, where all splice variants include a mutually exclusive exon, either A or B in order to maintain an open reading frame (Kofuji et al., 1994).

In general, excitable tissues contain exon A, whereas non-excitable tissues comprise NCX with exon B (Quednau et al., 1997). The cardiac (ACDEF), kidney (BD), and brain (AD) splice variants exhibit distinct properties for Ca^2+^-dependent allosteric regulation of NCX activity, thereby suggesting that exon-dependent regulatory properties may have physiological relevance (Matsuoka et al., 1995; Dyck et al., 1999). For example, cytosolic Ca^2+^ elevation activates the brain, cardiac, and kidney splice variants, whereas Ca^2+^-induced alleviation of Na^+^-dependent inactivation is observed only in the cardiac and brain splice variants (containing exon A) (Matsuoka et al., 1995; Dyck et al., 1999). The lack of significant Na^+^-transients in non-excitable tissues and their presence in excitable tissues explains the need for Ca^2+^-dependent alleviation of Na^+^-dependent inactivation only in excitable tissues. Although the cardiac (ACDEF) and brain (AD) variants exhibit similar regulatory responses to Ca^2+^, they differ in their response kinetics, with the kinetics of NCX1-ACDEF being ~ 10-fold slower compared with NCX1-AD (Matsuoka et al., 1995; Dyck et al., 1999). The kinetic differences are consistent with the slower Ca^2+^ transients involved in muscle contraction compared to the faster Ca^2+^ transients involved in neurotransmission (Berridge et al., 2003).

### Anomalous regulation of CALX

CALX1 is a *drosophila melanogaster* NCX ortholog, having a structure similar to that of mammalian NCXs (Schwarz and Benzer, 1997). However, electrophysiological characterization of the CALX1.1 splice variant revealed that despite having many properties common with NCX, it exhibits an opposite response to regulatory Ca^2+^ (Hryshko et al., 1996). That is, a rise in [Ca^2+^]_i_ (over the same range that activates NCX) inactivates CALX1.1 (Figure 2B). In the second variant, CALX1.2, Ca^2+^ has no regulatory effect on exchange activity (Omelchenko et al., 1998). CALX1 also undergoes alternative splicing only at CBD2, with its two variants differing only by five amino acids. The splicing region is at a position similar to that of the cassette exons in mammalian NCX1. The regulatory differences between CALX1 and NCX are especially interesting in light of the structural similarities between the regulatory CBDs among the different orthologs (see below).

## Structural and functional features of the regulatory cytosolic F-loop

**Figure 3:**
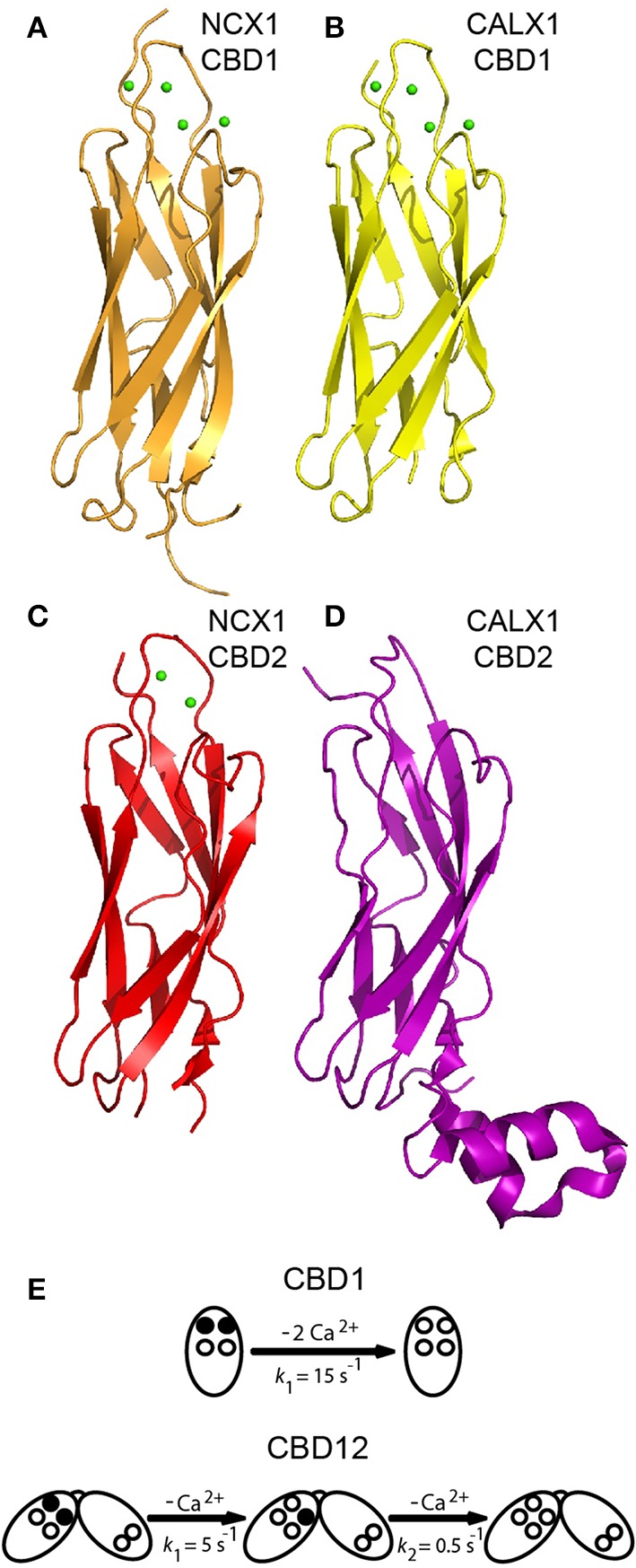
**Structures of isolated CBDs and dissociation kinetics**. Crystal structures of CBD1 from NCX1 (PDB 2DPK) **(A)**, CBD1 from CALX1 (PDB 3EAD) **(B)**, CBD2 from NCX1-AD (PDB 2QVM) **(C)**, and CBD2 from CALX1.1 (PDB 3E9U) **(D)** in cartoon representation. **(E)** Dissociation kinetics of two Ca^2+^ ions from the Ca3-Ca4 sites of isolated CBD1 and CBD12. The Ca3-Ca4 sites occupied by Ca^2+^ ions are denoted by filled circles, whereas the open circles represent empty Ca^2+^ sites. The indicated *k*_*off*_ values represent typical values observed in stopped-flow experiments.

**Figure 4:**
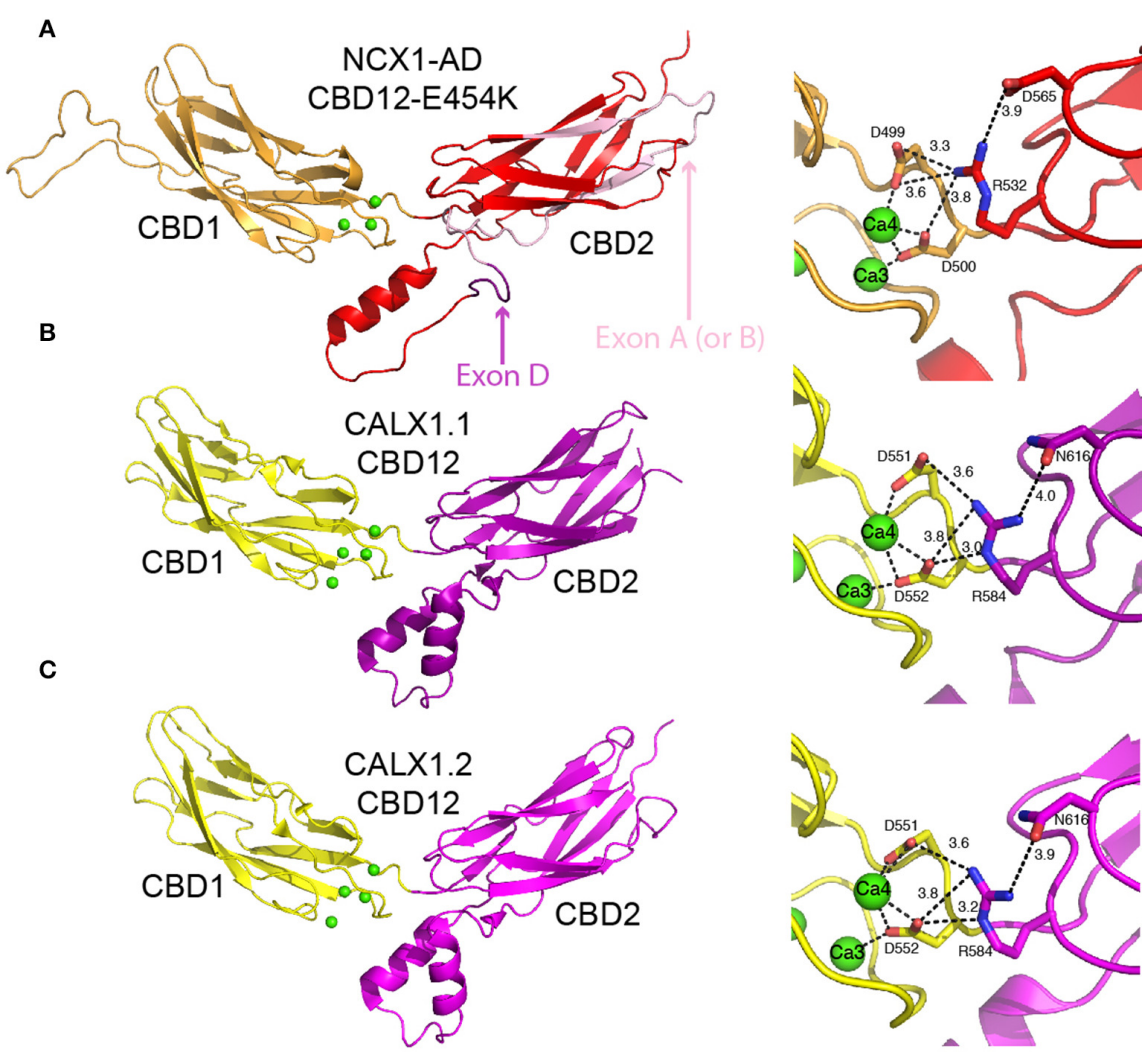
**Crystal structures of CBD12**. Crystal structures of CBD12-E454K from NCX1-AD (PDB 3US9) **(A)**, CBD12 from CALX1.1 (PDB 3RB5) **(B)**, and CBD12 from CALX1.2 (PDB 3RB7) **(C)** in cartoon representation. Residues participating in the network of interdomain salt bridges are represented as sticks to the right of each structure, with the bond distances within the network indicated. In **(A)**, missing loops were constructed using MODELER. The region corresponding to the mutually exclusive exon is pink, whereas the cassette exon is purple, as indicated by the arrows.

All eukaryotic NCX orthologs (including CALX1 from *Drosophila melanogaster*, which exhibits an anomalous regulation) contain the large cytosolic f-loop between TM5 and TM6. At the N-terminus, adjacent to the membrane, is an amphipathic α-helical region (20 residues) termed XIP (eXchanger Inhibitory Peptide) because it inhibits NCX when applied exogenously (Li et al., 1991). This area has been implicated in regulation of intact NCX by Na^+^ and phospholipids (Matsuoka et al., 1997). The XIP region is followed by a sequence predicted to be an α-helical Catenin-Like Domain (CLD) (Hilge et al., 2006), although this prediction has not been experimentally validated to date. Two consecutive CBDs are located downstream of the presumed CLD (Hilge et al., 2006), serving as sensors for regulatory Ca^2+^. CBD1 and CBD2 (Figures 3A–D) are connected in a head-to-tail fashion through a very short linker (five residues) that forms a two-domain (CBD12) regulatory tandem (Figure 4) (Hilge et al., 2006; Giladi et al., 2012c). This arrangement appears to be a crucial factor for governing the structure-dynamic interactions between the two domains, which definitely has functional significance in terms of decoding and propagation of the allosteric signal upon Ca^2+^ binding to the primary allosteric sensor at CBD1 (Giladi et al., 2010, 2012c). Importantly, the alternatively spliced region of NCX is exclusively located in CBD2 (Hilge et al., 2006; Hilge, 2012).

### Structures of isolated CBD1 and CBD2

High-resolution X-ray and NMR structures of isolated CBD1 and CBD2 from NCX1 revealed that each domain exhibits an immunoglobulin-like β-sandwich structure with seven antiparallel β-strands (Figures 3A,C) (Hilge et al., 2006; Nicoll et al., 2006; Besserer et al., 2007). The domains are nearly identical structurally, with a root mean square deviation of 1.3 Å. The Ca^2+^ binding sites are located at the C-terminal region of the domains. CBD1 contains four Ca^2+^ binding sites (Ca1-Ca4); the brain splice variant (AD) of CBD2 contains two Ca^2+^ binding sites (CaI-CaII) (Nicoll et al., 2006; Besserer et al., 2007). In contrast, the kidney splice variant (BD) of CBD2 does not bind Ca^2+^ (Hilge et al., 2009). The differences in CBD2 Ca^2+^ binding capacity result from the fact that exons A and B encode strands E-F of CBD2, which form part of the ion-binding region. The cassette exons (C, D, E, and F) are positioned at the N-terminal portion of CBD2's F-G loop, adjacent to the CBD1 binding sites (Figure 4A), and thus do not affect the CBD2 Ca^2+^ binding sites (Hilge et al., 2006, 2009; Giladi et al., 2012c). Ca^2+^ binding to CBD1 (which does not undergo alternative splicing), specifically to the Ca3-Ca4 sites, results in NCX activation (Ottolia et al., 2009). The alleviation of Na^+^-dependent inactivation depends on Ca^2+^ binding to the CaI site of CBD2 (and thus variants containing B-exon cannot relieve Na^+^-dependent inactivation, as mentioned above) (Besserer et al., 2007; Ottolia et al., 2009). NMR and crystallographic analyses of the Ca^2+^-bound and—free forms have shown that CBD2-AD retains its structural integrity in the absence of Ca^2+^ (Hilge et al., 2006; Besserer et al., 2007). In contrast, CBD1's binding sites become unstructured in the absence of Ca^2+^, whereas the core of the domain retains its structure and dynamics (Hilge et al., 2006). This difference arises from the presence of K585 in CBD2, in a position homologous to E454 in CBD1. In the absence of Ca^2+^, K585 forms salt bridges with negatively charged Ca^2+^ coordinating residues to stabilize CBD2's binding sites in the apo form (Besserer et al., 2007).

X-ray structures of isolated CBD1 and CBD2 from CALX (Figures 3B,D) revealed that they are highly similar to the NCX1 domains (Wu et al., 2009, 2010). CALX1-CBD1 binds four Ca^2+^ ions similarly to NCX1-CBD1, whereas CALX1-CBD2 does not bind Ca^2+^. Most of the FG loop is unstructured in NCX-CBD2, except for a short α-helical region in the C-terminal portion of the FG loop (Hilge et al., 2006). In contrast, in CALX-CBD2 the FG loop is organized as two helices perpendicular to the β-sheets (Wu et al., 2009). Owing to the high structural similarity, the different regulatory responses of CALX1 and NCX1 to changes in [Ca^2+^]_i_ cannot be attributed to structural differences between the isolated CBDs.

### Kinetic and equilibrium properties of Ca^2+^ binding to isolated CBDs

Isolated CBD1 and CBD2 exhibit distinct Ca^2+^ binding properties (Hilge et al., 2006; Boyman et al., 2009). The equilibrium binding constants (K_d_s) were measured in our laboratory by ^45^Ca^2+^ equilibrium binding assays and the rate constants of Ca^2+^ dissociation were measured using stopped-flow kinetics (Boyman et al., 2009). In both NCX1 and CALX1, CBD1 binds two Ca^2+^ ions with high affinity (K_d_ ~ 0.2 μM) at its Ca3-Ca4 sites and two Ca^2+^ ions with lower affinity (>5–10 μM) at its Ca1-Ca2 sites. Monophasic dissociation of two Ca^2+^ ions from the Ca3-Ca4 sites is observed in both NCX1 and CALX1, with a rate constant of ~15 s^−1^ (Figure 3E) (Giladi et al., 2012a). Ca^2+^ dissociation from Ca1-Ca2 is too rapid to be measured using stopped-flow kinetics, since the rate constant is >300 s^−1^ (Boyman et al., 2009). NCX1-CBD2-AD binds two Ca^2+^ ions: one with moderate affinity (K_d_ ~ 5 μM) at its CaI site and one with low affinity (K_d_ > 20 μM) at its CaII site. Ca^2+^ dissociates from CaI with a rate constant of ~ 150 s^−1^. As in CBD1, the dissociation rate constant from the low-affinity CaII site is too fast to be measured using stopped-flow kinetics (Boyman et al., 2009). As mentioned above, NCX1-CBD2-BD and both the CALX1-CBD2 splice variants do not bind Ca^2+^ (Giladi et al., 2012a). The K_d_ values measured for the Ca3-Ca4 sites of CBD1 match the K_0.5_ value for the “peak-current” activation of NCX, and the K_d_ value of CaI matches the K_0.5_ value for steady-state activation (and alleviation of Na^+^-dependent inactivation) (Hilgemann et al., 1992a; Ottolia et al., 2009), thus supporting the role of each domain in allosteric NCX regulation. However, the Ca^2+^ dissociation rate constants from isolated CBD1 and CBD2 cannot represent the slow I_2_ inactivation observed upon the removal of Ca^2+^from the intracellular surface of NCX1, occurring over several seconds (Hilgemann et al., 1992a).

### CBDs interact with H^+^ and Mg^2+^, but not with Na^+^

Eukaryotic NCX is extremely sensitive to cytosolic acidification (a pH decrease from 7.2 to 6.9 results in nearly 90% inactivation of NCX), thus demonstrating the physiological relevance of the NCX “proton block” under acidosis and ischemia conditions (Boyman et al., 2011). In general, H^+^ may interact with the transport domains, although there is no evidence that within a physiological range of pHs the protons affect the ionization of the ion-binding transport sites. Recent studies in intact cardiomyocytes as well as on isolated preparations of CBD1, CBD2, and CBD12 proteins clearly demonstrated that Ca^2+^ and H^+^ can compete with each other for binding to the functional CBD sites (Boyman et al., 2011). Notably, the close adjacency of the Ca^2+^ sites in the CBDs is consistent with the sharp dependence of Ca^2+^ binding on pH, thereby suggesting the cooperative nature of binding domain folding. Namely, the binding of the first Ca^2+^ ion may partially (or fully) deprotonate one or more coordinating residues, thereby enabling the next Ca^2+^ ion to bind to the remaining sites. A similar mechanism was proposed for the C_2_ domain of phospholipase A2, in which two Ca^2+^ sites are separated by 4.1Å (Malmberg et al., 2004). The physiological significance of these findings is that acidic pH may shut down NCX in a very cooperative and effective way, and prevent NCX-mediated currents that impose a high risk for generating cardiac arrhythmias under ischemia/acidosis conditions.

In light of the fact that only three Ca^2+^ binding sites (Ca3, Ca4, and CaI) actually contribute to [Ca^2+^]-dependent regulation of full-size NCX1 in the cellar system (Besserer et al., 2007; Ottolia et al., 2009), it is reasonable to ask what is the functional role of the remaining three low-affinity sites (K_d_ > 20 μM) (Boyman et al., 2009). Most probably, the low-affinity sites (Ca1, Ca2, and CaII) are Mg^2+^ rather than Ca^2+^ binding sites, which are constitutively occupied by Mg^2+^ under physiologically relevant ionic conditions (Boyman et al., 2009; Breukels et al., 2011; Giladi et al., 2013). Interestingly, the occupation of the Ca1-Ca2 sites by Mg^2+^ decreases the affinity of the primary sensor (Ca3-Ca4 sites), whereas the occupation of the CaII site by Mg^2+^ increases the affinity of the CaI site (Boyman et al., 2009). The physiological significance of this could lie in keeping the primary and secondary Ca^2+^ sensors within a physiologically relevant range, thereby covering the effective concentration range of 0.2–10 μM Ca^2+^.

Since Na^+^-dependent regulation of NCX is abolished along with Ca^2+^- and H^+^- dependent regulation upon α-chymotrypsin treatment of NCX, one can hypothesize that Na^+^, H^+^, and Ca^2+^ compete over the same regulatory site. This possibility was examined by performing equilibrium Ca^2+^ binding and stopped-flow assays in buffers containing 100 mM choline chloride, 100 mM NaCl, or 100 mM KCl. No significant differences were observed in these experiments, excluding the possibility that Na^+^ directly affects Ca^2+^ binding to CBDs (Boyman et al., 2009).

### CBD interactions in the context of CBD12 markedly alter Ca^2+^ sensing

As mentioned above, Ca^2+^ dissociation kinetics from either isolated CBD1 or CBD2 cannot account for the slow I_2_-inactivation observed in intact NCX upon [Ca^2+^]_i_ removal. However, in the context of CBD12, the domains display markedly altered Ca^2+^ affinity and dissociation kinetics (Giladi et al., 2010). In NCX1-CBD12-AD, the CBD1 sites bind Ca^2+^ with ~7–10 higher affinity compared with that observed in isolated CBD1 (Boyman et al., 2011; Giladi et al., 2012a). Strikingly, Ca^2+^ dissociates from the CBD1 Ca3-Ca4 sites in a bi-phasic (and not monophasic) fashion, with a fast component (*k*_f_ ~ 5 s^−1^) and a slow component (*k*_s_ ~ 0.5 s^−1^) (Figure 3E) (Giladi et al., 2010). The slow component, representing the occlusion of one Ca^2+^ ion, is a hallmark of domain interactions and closely matches the I_2_-inactivation kinetics of NCX1-AD (Dyck et al., 1999). These interactions are dependent on the short interdomain linker, as either an elongation of the linker (by insertion of seven alanine residues) or the linker mutations abolish the domains' interactions (Giladi et al., 2010, 2012b). Similar observations were made for other NCX1-CBD12 splice variants (BD, ACDEF) and also for the CALX1-CBD12 splice variants (Hilge et al., 2009; Giladi et al., 2012a). However, alternative splicing of CBD2 modulates the domains' interactions, resulting in up to 10-fold differences in Ca^2+^ affinity and dissociation kinetics from CBD1 in the different CBD12 splice variants (Giladi et al., 2012a). These differences account for the differences in the I_2_-inactivation kinetics observed in intact NCX. Thus, domain interactions are common among NCX orthologs and splice variants, but they are modulated in an ortholog and splice-variant dependent manner to meet physiological requirements.

## Structural basis for the allosteric regulation of NCX proteins

Crucial mechanistic questions that have emerged from the studies described above are as follows: (i) how does Ca^2+^ binding couple with regulatory conformational transitions to decode the allosteric signal, (ii) how is the regulatory signal diversified by alternative splicing, and (iii) how does the coupling of domains contribute to the transmission of regulatory information to ion transport domains. These questions were addressed using a variety of structural approaches, which are discussed below.

### Crystal structures of CBD12 reveal the Ca^2+^-driven structural organization of a highly conserved two-domain interphase

As an initial step to characterize the domains' interactions, the coupling of Ca^2+^ binding to conformational transitions underlying allosteric regulation, and the role played by alternative splicing, the crystal structures of CBD12-E454K (a mutant of NCX1-CBD12-AD), CALX1.1-CBD12, and CALX1.2-CBD12 were determined (Figure 4) (Wu et al., 2011; Giladi et al., 2012c). Intriguingly, the structures show striking overall similarity despite the different regulatory responses of the corresponding exchangers. The interface has a fairly small surface area (~ 350 Å in CBD12-E454K), explaining the need for a short interdomain linker to allow the domains to interact (Giladi et al., 2012c). In NCX1, the most important feature of the interface is a network of salt bridges, centered at R532 from CBD2. R532 forms bifurcated salt bridges with D565 of CBD2, on the one hand, and with D499 and D500 at the Ca3-Ca4 sites of CBD1, on the other hand (Figure 4A). Importantly, D499 and D500 also coordinate Ca^2+^ at the Ca3-Ca4 sites. Thus, the interdomain salt bridges stabilize Ca^2+^ binding, resulting in Ca^2+^ occlusion at the interface. In return, Ca^2+^ binding to the Ca3-Ca4 sites stabilizes the interface, resulting in the coupling of Ca^2+^ binding to signal transmission through CBD2 to the membrane domains. This is supported by the fact that D499 and D500 are disordered in the apo form (Hilge et al., 2006), making Ca^2+^ binding essential for robust interdomain interactions. The structural role of Ca^2+^ is also suggested by the fact that the Ca3-Ca4 sites are completely buried in the interface. Finally, mutation of the central residue in the mentioned network, R532, abolishes Ca^2+^ occlusion and bi-phasic dissociation kinetics (Giladi et al., 2012c). Similar networks exist in CALX1.1 and CALX1.2, although an Asn residue is found in the position corresponding to D565 (Figures 4B,C).

Based on the crystal structures of the CALX1 splice variants, regulatory differences were attributed to the different hinge angles between the CBDs (118° and 110.5° for CALX1.1 and CALX1.2, respectively; Wu et al., 2011). However, this interpretation has been challenged by the crystal structure of CBD12-E454K, in which the hinge angle (117.4°) is nearly identical to that of CALX1.1 (Giladi et al., 2012c). These findings are especially interesting in the context of the regulatory differences in these variants, since NCX1 is activated by regulatory Ca^2+^, whereas CALX1.1 is inhibited by allosteric interactions with Ca^2+^ (Figure 2). Notably, the alternatively spliced region is not directly involved in the interface of either of the examined CBD12 constructs (Figure 4A). Thus, the structural similarities between CBD12 from NCX and CALX imply that the different responses to regulatory Ca^2+^ cannot be attributed solely to the CBDs' orientation in the CBD12 tandem.

The sequence conservation of the two-domain interface and the structural similarities between the mentioned structures point to a general mechanism for regulating the NCX family (Giladi et al., 2012c). Importantly, the architecture of this interface differs from that of tandem Ca^2+^-binding C_2_ domains (e.g., of synaptotagmin and PKC) (Stahelin et al., 2005), implying a different mode of action. However, CBD12 shares a striking similarity with the cadherin extracellular domain, which bears multiple β-sandwich domains bridged by small interfaces, and which contains three Ca^2+^ sites. Cadherin undergoes Ca^2+^-dependent rigidification (Häussinger et al., 2002), enabling cell-cell interactions. This supports the importance of stabilizing the domains' interactions by Ca^2+^ binding to the Ca3-Ca4 sites in the allosteric regulation of NCX. Nevertheless, the Ca^2+^ binding modes of cadherins and CBD12 are dissimilar. Namely, Ca^2+^ binding to the two-domain cadherin construct involves direct interactions with residues in the linker region, whereas the binding of Ca^2+^ to sites Ca3 and Ca4 in CBD1 involves ligation with residues 498–500 that directly precede the CBD12 linker.

### SAXS reveals a Ca^2+^-dependent population shift in CBD12

The crystallographic structures of CBD12 provided insight into the domains' interactions in the Ca^2+^-bound state in atomic details. However, it is merely a snapshot, although in high resolution, of a dynamic protein. To assess the effect of ligand binding on CBD12, we utilized SAXS, which provides time- and space-averaged information regarding the protein conformation in solution, although in low resolution (10–20 Å) (Bernadó et al., 2007; Blanchet and Svergun, 2013). The data were analyzed using the ensemble optimization method (EOM), which fits the average theoretical scattering intensity from an ensemble of possible conformations (selected from a pool of random conformations) suitable to the experimental SAXS data (Bernadó et al., 2007).

**Figure 5:**
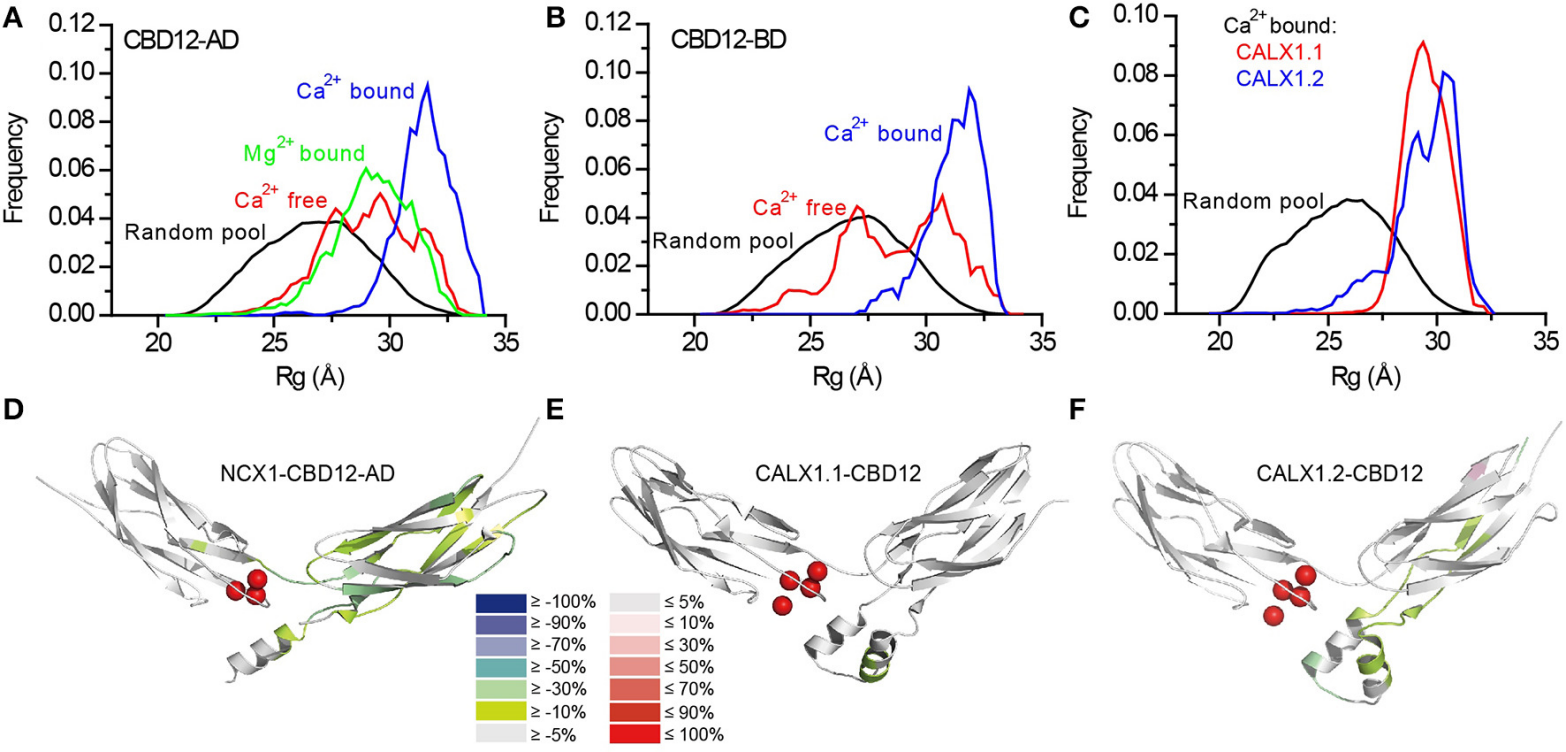
**SAXS-EOM analysis and HDX-MS of NCX orthologs and splice variants**. Random R_g_ pools and selected EOM ensemble distributions for CBD12-AD **(A)**, CBD12-BD **(B)**, and Ca^2+^-bound CALX1.1 and CALX1.2 CBD12 **(C)**. The difference between the HDX profiles of the apo and Ca^2+^-bound forms of NCX1-CBD12-AD at 10.000 s exchange time **(D)**, CALX1.1-CBD12 at 100 s exchange time **(E)**, and CALX1.2-CBD12 at 100s exchange time **(F)** are overlaid onto the crystal structures of the different CBD12 proteins (PDB 3US9, 3RB5, and 3RB7, respectively). Ca^2+^ is indicated as red spheres. The color legend shows the differential HDX after Ca^2+^ binding.

Two splice variants of NCX1-CBD12 (AD, BD) were examined (Giladi et al., 2013). Whereas the global structural parameters (e.g., the maximal intramolecular distance, the radius of gyration) were largely similar in the apo- and the Ca^2+^-bound forms, the EOM analysis revealed strikingly different conformational distributions (Figures 5A,B). That is, in the apo state CBD12 exhibits a wide range of conformations, whereas Ca^2+^ binding narrows the conformational distribution in line with a population-shift mechanism. According to these data, Ca^2+^ binding to the Ca3-Ca4 sites results in a population shift, where more constrained conformational states become highly populated at a dynamic equilibrium in the absence of global conformational transitions in CBD alignment (Giladi et al., 2013). This seems to be true for all the examined splice variants. In addition, the conformational distributions of CBD12 from CALX1.1 and CALX1.2 in the Ca^2+^-bound state are nearly identical to those of the NCX1-CBD12 tandems examined (Figure 5C). These results are in line with Ca^2+^-dependent domains' rigidification, and are in agreement with NMR studies of NCX1-CBD12-AD (Salinas et al., 2011). Notably, this is specifically a Ca^2+^-switch rather than an electrostatic switch, since Mg^2+^ cannot impose a population shift (Giladi et al., 2013). Moreover, a population shift is also observed in the CBD12-E454K mutant, which has partial charge neutralization in the apo form (Giladi et al., 2013).

The crystallographic and SAXS data presented thus far suggested common Ca^2+^ dependent interactions between the domains. Although these data are highly important for understanding the allosteric signal propagation between the domains, it remains unclear how the allosteric signal is diversified and propagated in the different splice variants and orthologs. Two possibilities were raised: (i) additional structural elements in the regulatory f-loop and/or membrane domain are involved in decoding and specifying the regulatory effects or alternatively, (ii) the conformational dynamics differ between the NCX splice variants and CALX, despite the similar orientations observed in the crystal structures and the SAXS-EOM data obtained for diverse splice variants, because there are either positive, negative, or no responses to regulatory Ca^2+^.

### HDX-MS reveals the structure-dynamic basis of diverse NCX regulation

To test the possibility that dynamic interactions between the two domains underlie the differential responses to regulatory Ca^2+^, CBD12 from NCX1-AD (positive response), CALX1.1 (negative response), and CALX1.2 (no response) were studied using the advanced approaches of HDX-MS (Giladi et al., 2015). In general, HDX-MS measures the exchange rates of peptide amide hydrogen with deuterium in the solvent. In folded proteins, the exchange rate varies, depending on the position of the amide hydrogen. The secondary structure, flexibility, and the dynamics of the protein conformation affect the deuterium uptake level. HDX was measured in the presence and absence of Ca^2+^ to study the structural outcomes of binding in the differentially regulated isoforms.

#### CBDs also interact in the absence of Ca^2+^

To study the domains' interactions in the apo form, HDX-MS was used to study NCX1-CBD12-AD and its mutant, CBD12-F450G. F450 is a central residue in the hydrophobic core of the domains' interface (Giladi et al., 2012c) and the F450G mutation results in the domains' uncoupling, as reflected by the decreased Ca^2+^ affinity and the lack of Ca^2+^ occlusion (Giladi et al., 2015). The HDX-MS analysis revealed that in the uncoupled mutant, CBD1 is less stable in the apo form compared with WT NCX1-CBD12-AD. Thus, the domains' interactions in the apo form stabilize CBD1; however, this stabilization is interrupted by the uncoupling effect of F450. This may explain some of the Ca^2+^ binding properties of CBD12. The domains' interactions result in ~50-fold slower dissociation kinetics of the occluded Ca^2+^ ion from CBD1, but the affinity is only ~7-fold increased, implying ~7-fold reduction in the association kinetics (K_d_ = *k*_*off*_*/k*_*on*_) (Giladi et al., 2010, 2012a; Boyman et al., 2011). As mentioned above, the isolated CBD1 binding sites are disordered in the absence of Ca^2+^ (Hilge et al., 2006; Wu et al., 2010). The stabilization of the apo form may reduce the Gibbs free energy (ΔG) for the disorder-to-order transition upon Ca^2+^ binding, thereby making the binding less favorable, due to the reduced values of *k*_*on*_.

#### Alternative splicing modifies the domains' coupling

Although the alternative splicing region of CBD2 is not directly involved in the interface, it clearly affects the domains' interactions as suggested by the equilibrium binding, kinetics, and HDX-MS assays (Giladi et al., 2012a, 2015). The helical region on CBD2, adjacent to the CBD1 binding sites, is similar for NCX1-CBD12-AD, and CALX1.2-CBD12, encompassing an additional turn as compared with CALX1.1-CBD12 (Figures 5D–F). The presence of this turn is thus dependent on the adjacent alternative splicing segment in the CALX1 splice variants. The additional helical turn exhibits reduced deuterium uptake upon Ca^2+^ binding in both NCX1-CBD12-AD (Figure 5D) and CALX1.2-CBD12 (Figure 5F) and the lack of this turn in CALX1.1-CBD12 may interfere with the stabilization of the interface by Ca^2+^ (Figure 5E). Thus, although not directly participating in the interface, the alternative-splicing segment can indirectly influence the domains' coupling, resulting in regulatory diversity.

#### Structural dynamics correlate with the regulatory response to Ca^2+^

In all the constructs examined by HDX-MS, Ca^2+^ binding resulted in backbone rigidification of CBD2, as reflected by a decreased deuterium uptake (Figures 5D–F). The rigidification of CBD2 cannot be fully attributed to Ca^2+^ binding at CBD2, since the CALX splice variants bind Ca^2+^ only at CBD1 (Wu et al., 2009; Giladi et al., 2012a). Moreover, the uncoupling of the F450G mutation at CBD1 results in less Ca^2+^-dependent rigidification of the main chain in CBD2. These results indicate that Ca^2+^ binding to CBD1 is sensed at CBD2. However, the extent and intensity of the Ca^2+^-induced rigidification occur at varying degrees in distinct splice variants (Figures 5D–F). Most importantly, the Ca^2+^-induced decrease in HDX at CBD2 upon Ca^2+^ binding correlates with regulatory specificity (negative, positive, or no response to Ca^2+^) in a given splice variant. For CALX1.1-CBD12, in which a minimal response to Ca^2+^ occurs (Figure 5E), the exchanger remains inhibited. For NCX1-CBD12-AD, in which the maximal response to Ca^2+^ occurs (Figure 5D), the exchanger is activated; an intermediate phenotype (no response) is observed for CALX1.2-CBD12 (Figure 5E), which also exhibits intermediate HDX changes in response to Ca^2+^. These data support the notion that the stabilization of CBD2 dynamics is involved in allosteric regulation in a splice variant-dependent manner. Further HDX-MS studies using NCX-CBD12 isoforms and splice variants will delineate the specific roles of individual exons in tissue-specific splice variants of NCX (Khananshvili, 2013, 2014).

## Implications for NCX-specific drug design

Since NCX participates in numerous physiological and pathophysiological processes (Blaustein and Lederer, 1999; Lytton, 2007; Khananshvili, 2013), developing specific drugs for NCX variants is highly desired. However, drugs that directly affect NCX are not currently clinically available. The major structural advancements described above may facilitate the development of appropriate drug candidates. The crystal structures of CBD12 can provide a framework for structure-based computational screening, in which small molecules are ranked on the basis of docking to protein structures (Kitchen et al., 2004; Taboureau et al., 2012). This method allows the screening of enormous compound databases. Drugs targeting the CBDs, rather than the ion translocation sites, have the potential to efficiently target tissue-specific NCX variants since the alternative splicing region of NCX lies within CBD2 (Hilge et al., 2006). More specifically, drugs targeting the domains' interface, adjacent to the alternative splicing region, are of particular interest. These drugs can potentially enhance NCX activity via domain stabilization or inhibit NCX by disrupting the domains' interactions. The stabilizing or destabilizing effects of specific compounds can be further tested using the structural methods described above (SAXS-EOM, HDX-MS).

## Conclusions

Recent structural and biophysical studies have shed light on the structural basis of ion transport and the allosteric regulation of NCX proteins. The structure of NCX_Mj (Liao et al., 2012), along with MD simulations and ion-flux analyses (Marinelli et al., 2014), verified the exchange mechanism and stoichiometry and provided important clues regarding the molecular basis of NCX ion selectivity (Figure 1). Structural and biochemical studies of the regulatory CBD12 tandem by a variety of techniques revealed some features that are common among all NCX orthologs and splice variants (Giladi et al., 2012c, 2013). These common features can be modulated in different NCX orthologs, isoforms, and splice variants to meet tissue-specific physiological demands (Giladi et al., 2012a, 2015). CBD1 and CBD2 interact in the context of CBD12 (Figure 4; Giladi et al., 2010), resulting in the increased affinity of the CBD1 binding sites and in Ca^2+^ occlusion (Figure 3E) (Boyman et al., 2011; Giladi et al., 2012a); the extent of these effects depends on the specific ortholog or splice variant examined (Figures 5D–F). Common and conserved interdomain interactions underlie this phenomenon, as demonstrated by X-ray crystallography and SAXS (Figures 4, 5A–C; Giladi et al., 2012c, 2013). The binding of Ca^2+^ to the primary sensor (Ca3-Ca4 sites) in CBD1 rigidifies CBD12, whereas the domains' interactions in turn stabilize Ca^2+^ binding, resulting in Ca^2+^-dependent regulation. This represents a common mechanism for decoding the initial information upon Ca^2+^ binding for all NCX isoform/splice variants. The CaI site on CBD2 exhibits structural variances, while being responsible for the Ca^2+^-dependent alleviation of Na^+^-dependent inactivation (Hilge et al., 2009). The structural basis for the diverse regulatory responses to Ca^2+^ binding in different orthologs and splice variants is related to the extent and strength of CBD2 rigidification upon Ca^2+^ binding to CBD1 (Figures 5D–F; Giladi et al., 2015). It is hoped that breakthroughs in understanding the structure-function relationships in NCX proteins will allow for the future pharmaceutical development of tissue-selective NCX-directed drugs.

## Author contributions

All authors listed, have made substantial, direct and intellectual contribution to the work, and approved it for publication.

### Conflict of interest statement

The authors declare that the research was conducted in the absence of any commercial or financial relationships that could be construed as a potential conflict of interest.
